# Culture Medium Development for Microbial-Derived Surfactants Production—An Overview

**DOI:** 10.3390/molecules23051049

**Published:** 2018-05-01

**Authors:** Abdul Hamid Nurfarahin, Mohd Shamzi Mohamed, Lai Yee Phang

**Affiliations:** 1 Department of Bioprocess Technology, Faculty of Biotechnology and Biomolecular Sciences, Universiti Putra Malaysia, Serdang, Selangor 43400 UPM, Malaysia; nurfarahinabdulhamid93@yahoo.com (A.H.N.); m_shamzi@upm.edu.my (M.S.M.); 2 Bioprocessing and Biomanufacturing Research Centre, Faculty of Biotechnology and Biomolecular Sciences, Universiti Putra Malaysia, Serdang, Selangor 43400 UPM, Malaysia

**Keywords:** biosurfactants, culture medium development, carbon sources, nitrogen sources, C/N ratio, minerals, trace elements

## Abstract

Surfactants are compounds that can reduce the surface tension between two different phases or the interfacial tension of the liquid between water and oil, possessing both hydrophilic and hydrophobic moieties. Biosurfactants have traits that have proven to be advantageous over synthetic surfactants, but these compounds do not compete economically with synthetic surfactants. Different alternatives increase the yield of biosurfactants; development of an economical production process and the usage of cheaper substrates during process have been employed. One of the solutions relies on the suitable formulation of a production medium by including alternative raw materials sourced from agro-wastes, hydrocarbons, or by-products of a process might help in boosting the biosurfactant production. Since the nutritional factors required will be different among microorganisms, the establishment of a suitable formulation for biosurfactant production will be challenging. The present review describes various nutrients and elements considered in the formulation of a production medium with an approach focusing on the macronutrient (carbon, nitrogen source, and C/N ratio), minerals, vitamins, metabolic regulators, and salinity levels which may aid in the study of biosurfactant production in the future.

## 1. Introduction

Surfactants are amphipathic molecules that lower the surface tension of a liquid or the interfacial tension between two liquids, such as that of oil and water. They are characteristically organic compounds containing both hydrophobic tails and hydrophilic heads [1]. Surfactants can be synthetic or biological in origin. For over several decades, surfactants are chemically synthesized and classified according to their dissociation level in water. There can be anionic, cationic, and non-ionic surfactants. Good performing synthetic surfactants are called Gemini or dimeric surfactants which are composed of two hydrophobic chains and two hydrophilic moieties linked by a spacer group that can be hydrophobic/hydrophilic and flexible/rigid [2,3]. Microbial-derived surfactants (or in short, biosurfactants), on the other hand, are those being naturally produced by microorganisms either constitutively or inducibly. A biosurfactant is an amphipathic molecule that can be classified into two different classes, low and high molecular weight biosurfactants. Low molecular weight biosurfactants are generally glycolipids, such as rhamnolipids or lipopeptides, whereas the high molecular weight biosurfactants encompass amphipathic polysaccharides, lipopolysaccharides, proteins, and lipoproteins. More attention has been paid to biosurfactant production in recent times due to the perceived advantages over synthetic surfactants, chiefly, their ease of biodegradation which significantly reduced the environmental burden in contrast to chemical processing, ecological adaptability which helps in many bioremediation processes, low toxicity which made them appealing for uses in the pharmaceutical, cosmetic [4], and food industry and high specificity that allow them to promote detoxification of selected pollutants under extreme conditions [5].

Biosurfactant-producing microorganisms are able to produce biosurfactants in aqueous media with the addition of carbon sources like glucose, fructose, glycerol [6], mannitol, and olive oil [7,8]. Biosurfactant produced into the culture medium acts to ease the movement of insoluble substrates across the cell membrane for the growth of microorganisms [9]. In other instances, it helps to stabilize oil or hydrocarbon in water and vice versa. For example, biosurfactants can greatly reduce the surface tension of water from 72 mN/m to 22 mN/m [10]. Owing to these properties, biosurfactants can solve some of the problems involving oil spill in oceans and enhance biodegradation of polycyclic aromatic hydrocarbon (PAH) compounds [11]. Biosurfactants also play important roles in several industries; in agricultural, biosurfactants help to improve plant growth by getting rid of phytopathogens [12] whereas, in the pharmaceutical industry, it can ease the introduction of foreign genes to the selected cells during gene therapy [13]. Likewise, in the food industry, they can serve as an emulsifier in confectionery production or solubilizer in foods containing fats and oils, such as margarine and dairy foods [14]. Due to the various applications served by biosurfactants, it is impossible to deny the high demand for them which had generated almost US$ 24 million in revenues in 2009 and is predicted to reach $2.8 billion in 2023 [15].

The problems in biosurfactant production mainly arises from costly substrates, where the likes of pure glucose were used in the process, low end-product titer, and formation of a variety of by-products as opposed to a singular desired biosurfactant leading to a ramping up in price [16]. Besides that, foam resulting from emulsification of biosurfactants due to vigorous agitation during cultivation might decrease the transfer efficiency of oxygen into the liquid medium [17] in which countermeasures can be taken to eliminate foaming but will further drive-up the production cost. To overcome these problems, various researchers were looking into cheaper alternative substrates, optimization of the environmental conditions, and improvement on the downstream recovery processes [18,19,20,21]. Still, no definitive method could contribute to the overall cost saving for biosurfactant production. It is opined here that the nutritional factors considered during the formulation of growth and production medium for any given biosurfactant-producing microbes are the key focus area to be scrutinized before other operating parameters come into the picture since they constitute about 30–40% of the total production cost at industrial scale [22]. Formulation of an optimized production medium comprised of a selection of proper nutrients at correct levels to give an ideal microenvironment for supporting growth and metabolite production [23]. The objective of this review is to provide an insight into different nutritional factors involved in the formulation of a production medium, specifically for biosurfactants production by various microorganisms so that some guidelines on the suitable medium formulation can be established. Overall, this review aims to provide the rationale for the selection and formulation of certain media components which may potentially improve the biosurfactants yield when executing a fermentation process.

## 2. General Culture Medium Development for Cultivation Process

Microorganisms basically need carbon sources, nitrogen sources, minerals, vitamins, growth factors, and water for them to propagate and form products. Logic dictates that it is far easier to develop culture media for a small-scale operation, simply by introducing pure compounds required for the growth of microorganisms. Nonetheless, the same media might not be good enough for production scale. The culture medium should exhibit significant properties such as permitting high selectivity toward target products over the undesired ones, can be disinfected easily, capable of generating a persistent product, accordant enough with different modes of cultivation, and does not generate any harmful waste along the cultivation process [24].

On a larger scale cultivation, the nutrients should come from relatively cheap sources, which should satisfy as many as possible the following criteria: the medium employed should offer the highest concentration or yield of biomass or product (per gram of substrate basis); enable maximum rate of product formation; provide minimum output of unwanted product; be economically accessible throughout the year; and induce the least difficulty in every level of cultivation, i.e., production, extraction, purification, and waste treatment [25]. Currently, agricultural waste like sugarcane molasses [26] and corn steep liquor [27] for carbon source, and urea or part of cultivation leftover as nitrogen source have the potential to be the cheap and abundant substrates to fulfill some of the above criteria. The very first step to consider in formulating the production medium is the stoichiometric balance based on the cell and product generation as in Equation (1):

Carbon & Energy source + Nitrogen source + Heat + Nutrients **→** Carbon Dioxide + Water + Biomass + Products (1)

**Figure 1:**
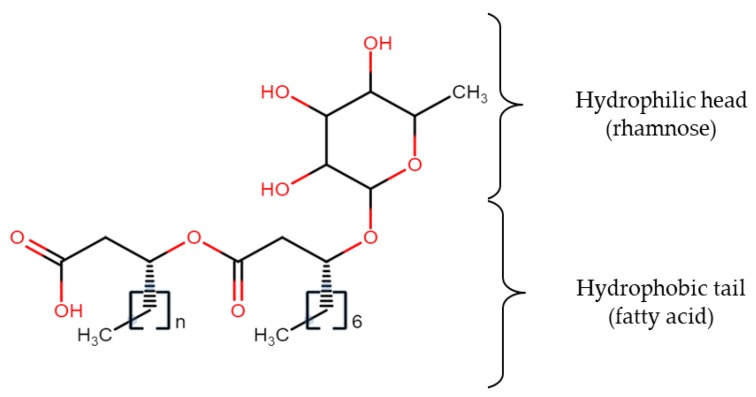
Biosurfactant molecule (rhamnolipid) with hydrophilic head and hydrophobic tail [31].

A well-known biosurfactant, rhamnolipid (Figure 1), contains one or two molecules of rhamnose (hydrophilic head) and β-hydroxy fatty acid (hydrophobic tail) linked together via a glycosidic bond. The most commonly found structures of rhamnolipids are monorhamnolipids and dirhamnolipids [28] while the lipid moiety may consist of hydroxyoctanoic acid (C_8_), hydroxydodecanoic acid (C_12_), and hydroxydodecenoic acid (C_12:1_) [29]. The main function of these low-molecular-weight biosurfactants is to reduce the surface and interfacial tensions, while high molecular weight biosurfactants are more effective at stabilizing oil-water emulsions [30]. The establishment of a suitable medium for biosurfactant production might be challenging since different microorganisms require different nutrients fed with different quantities, and medium design should also take into account the metabolic pathway of the particular microorganism as well as factoring the sugar and lipid moieties in the chemical structure. For instance, production of a biosurfactant can be enhanced by using inducer substrate like olive oil together with the main substrate. The other way is by employing the appropriate amount of chelating agent in the medium to help in solubilizing the iron component, so that inhibition of some metabolic pathways of certain microorganisms having high-salt intolerance can be avoided. The following part of the review will specifically discuss metabolic pathways involved in utilizing different types of carbon sources and various nutritional factors included in the medium for biosurfactant production that should be considered before initiating any cultivation process.

## 3. Metabolic Pathways of Biosurfactant Production

**Figure 2:**
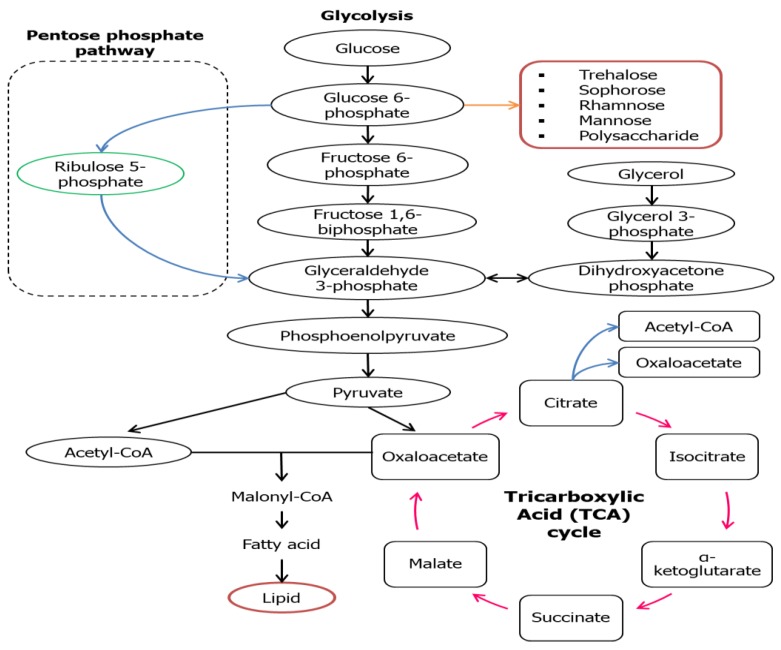
Metabolic pathways involved in synthesis of biosurfactants using water-soluble substrate [36,37].

For the microorganisms to grow and produce a hydrophilic moiety of biosurfactant, water-soluble substrates like carbohydrate groups are usually being utilized, while hydrophobic substrates like fats and oils are used to build up the hydrophobic portion of biosurfactants [32,33]. Varieties of metabolic pathways associated with the production of precursors of biosurfactant production are carbon sources that are nature-dependent that can be found in the culture medium. For example, the carbon flow will be regulated by both lipogenic pathway (lipid generation) and the development of hydrophilic moiety via glycolytic pathway, that are restrained by microbial metabolism (Figure 2) [34]. A water-soluble substrate like glucose is broken down to the intermediate; glucose-6-phosphate (G6P) via glycolytic pathway and this intermediate is one of the major precursors of carbohydrates found in the hydrophilic part of a biosurfactant. A series of enzymes are used to catalyze G6P on route to synthesizes various forms of hydrophilic moieties in the biosurfactant; trehalose, sophorose, rhamnose, mannose, and polysaccharide. For the formation of the hydrophobic moiety (lipid), glucose is oxidized to pyruvate which then transformed into acetyl-CoA that synthesizes malonyl-CoA when combined with oxaloacetate. Oxaloacetate will then be converted into fatty acid (precursors) for lipid production [35]. 

**Figure 3:**
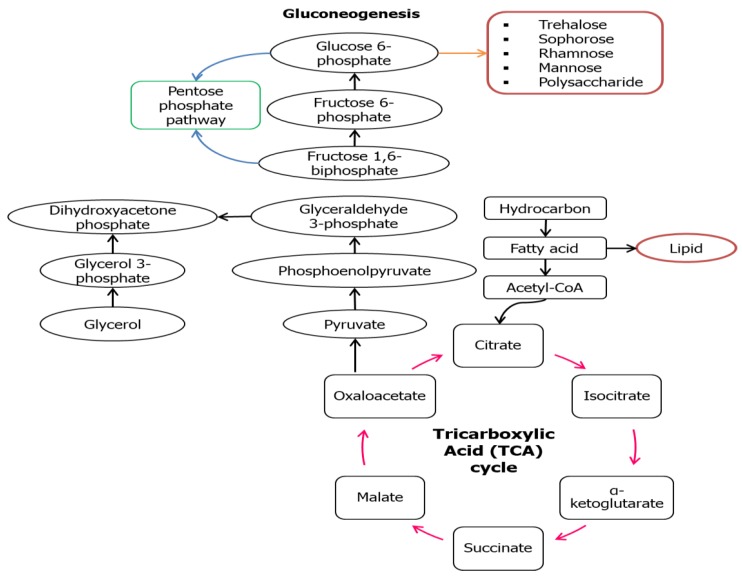
Metabolic pathways involved in synthesis of biosurfactant using hydrocarbon substrate [39,40].

However, in some cases like *Lactobacillus delbrueckii* N2, *L. cellobiosus* TM1, and *L. plantarum* G88, biosurfactants with higher lipid content were produced when using glycerol as the substrate compared to sugar cane molasses [38]. This might be due to the fact that these microbes could be mainly directed to the lipogenic pathway and gluconeogenesis (GNG) (Figure 3) that are usually being utilized by a hydrocarbon substrate. In the situation where hydrocarbon groups are used as the carbon source, the microbial mechanism might switch into the lipolytic pathway and GNG which leads to the hydrophobic moiety production and hydrophilic moiety will be produced de novo through GNG. The GNG pathway seems to be the reverse version of glycolysis to produce glucose as the end-product, but the reactions catalyzed by hexokinase, pyruvate kinase, and phosphofructokinase-1 are irreversible. GNG begins with the oxidation of fatty acids to form acetyl-CoA through β-oxidation that later will enter the tricarboxylic acid TCA cycle to form pyruvate. Conversion of pyruvate into polysaccharide precursors (G6P) involved series of enzymes similar in glycolysis except those mentioned three. Figure 3 illustrates the main reaction involved in the synthesis of both the hydrophilic and hydrophobic moiety in a biosurfactant using hydrocarbon as the substrate.

According to Wang et al. [41], the mechanisms of biosurfactant production and cell surface hydrophobicity can be altered according to the chain length of the alkanes used as the carbon source in biosurfactant production. Upon growth on hexadecane (C_16_), tetracosane (C_24_), and hexatriacontane (C_36_), *Dietzia* sp. strain DQ12-45-1b produced glycolipids, phospholipids, and lipopeptides, respectively. Interestingly, cultivation with C_36_ increased cell surface hydrophobic activity, which attenuated the negative effect of the decline of the emulsification activity. Generally, microorganisms are susceptible to hydrocarbon groups as the following rank: linear alkanes > branched alkanes > small aromatics > cyclic alkanes [42]. *Acinetobacter* sp. was found to be capable of utilizing alkanes of chain length C_10_–C_40_ as a sole source of carbon [43]. More carbon sources used in biosurfactant production were discussed in the following part of this review.

Last but not least, some multienzyme complexes are required after the production of lipid and sugar moieties in order to complete the process of biosurfactant formation. Among all the biosurfactants reported to date, the molecular biosynthetic regulation for rhamnolipid by *Pseudomonas aeruginosa* and surfactin synthesized by *Bacillus subtilis* were among the earliest biosurfactants to be decoded. According to Burger et al. [44], rhamnolipids production is carried out by two sequential glycosyl transfer reactions, each is catalyzed by a different rhamnosyltransferase while surfactins production is catalyzed non-ribosomally by a large multienzyme peptide synthetase complex called the surfactin synthetase [45]. In the case of lipopeptides, they are generally synthesized in a ribosome-independent manner with nonribosomal peptide synthetases [46].

## 4. Media Components for Biosurfactant Production

### 4.1. Carbon Sources

Biosurfactants producing microorganisms are usually heterotrophs whereby they consume organic constituent of carbon source to grow and produce their metabolites. About 30–40% of the total cost comes solely from the preparation of growth and production medium [17] for biosurfactant production, thus warranting the need for cheaper type of feedstocks. The biomass and product formation are usually being controlled by the carbon consumption rate of microorganisms during cultivation [47]. Generally, there are three types of carbon sources being commonly used in biosurfactant productions; carbohydrate, oils and fats, and hydrocarbon groups.

Under the carbohydrate group, simple sugar, starch, and plant sugar-based carbohydrates are the major carbon sources used in biosurfactant production. Glucose is the typical example of the carbon source which can easily be metabolized by microorganisms through the glycolysis pathway for the generation of energy and is commonly reported to give higher yield of product. Previous investigations on the carbohydrate group as the carbon source for biosurfactant production by various microorganisms is tabulated in Table 1.

*P. aeruginosa* MTCC 7815 utilized glucose much better than other carbon sources (glycerol, fructose, and starch) to yield higher amounts of biosurfactant, biomass, emulsification index, E24 (76.77%), and the lowest surface tension (34.53 mN/m) [51]. For exceptional biosurfactant producers like *Klebsiella* sp. RJ-03, production of biosurfactant was found to be the highest with starch followed by sucrose, xylose, galactose, glucose, and fructose [60]. In certain cases, different raw materials containing a variety of carbohydrate groups were tested on several microorganisms to produce biosurfactants. For example, a mineral medium containing clarified cashew apple juice (MM-CCAJ) which contained about 12.05 g/L of sugar [61] was utilized by *B. subtilis* LAMI005 to produce two-fold less than the amount produced using mineral medium (MM) supplemented with 10 g/L of glucose and 8.7 g/L of fructose (MM-GF) [62]. However, critical micelle concentration (CMC) of the biosurfactants produced using MM-CCAJ was 2.5-fold lower than the one produced using MM-GF, which indicates it is a more efficient biosurfactant and indicate that it is feasible to produce surfactin from clarified cashew apple juice. 

In normal cases, microorganisms will metabolize sugar substrate through the glycolysis pathway. Substrates like glycerol feed into the central carbon metabolism at the level of glyceraldehyde-3-phosphate and thus, do not employ the pentose phosphate (PP) pathway, which wastes the carbon via CO_2_ production. For glucose and sucrose, they will enter the central carbon metabolism via the Entner-Doudoroff (ED) pathway. The PP pathway will be activated when energy for cell maintenance is required (via redox cofactor synthesis) resulting in the loss of carbon through CO_2_ generation. When high amount of energy is required (i.e., high growth rates), full oxidation through acetyl-CoA and tricarboxylic acid (TCA) cycle happened and cause the CO_2_ formation which contributes to the lower biosurfactant (rhamnolipid) yield [63].

Utilization of various types of oils and fats as the carbon source in biosurfactant production has been well documented. This is due to the fact that the bioprocess pathway to manufacture biosurfactant itself undergoes four possibilities. First, the hydrophilic and hydrophobic parts of biosurfactants are developed de novo along independent pathways. Second, both of the biosurfactant moiety productions are influenced by the carbon source. Third, the hydrophilic part is synthesized de novo and the carbon substrate will promote the production of the hydrophobic part or lastly, the hydrophobic part is synthesized de novo and the carbon source will induce the production of the hydrophilic part.

Oil substrates have been sourced from fresh feedstock or waste by-product of a particular process. For example, *P. aeruginosa* D strain utilized 2% waste frying coconut oil effectively to produce 3.55 g/L of biosurfactant [17]. Other than that, *P. aeruginosa* F23 also utilized fresh coconut oil (2%) as carbon source to produce 2.8 g/L of biosurfactant and reduce the surface tension of medium from 45 mN/m to 31 mN/m when grown in an optimized SM medium [64]. *P. aeruginosa* achieved higher biosurfactant production when using waste frying coconut oil compare to fresh coconut oil in complex production medium due to higher free fatty acid content in waste frying coconut oil which supports the growth of microorganisms [65].

In the case of the fat substrate, the cultivation of *C. glabrata* UCP1002 yeast supplied with 5% vegetable fat waste in 100 mL aqueous medium ultimately produced a maximum value of 7 g/L biosurfactant after 144 h of incubation time [66]. This substrate yielded two times the biosurfactant compared to the run which used waste frying coconut oil. Alternatively, Santos et al. [67] evaluation on *C. lipolytica* UCP0988 ability to produce glycolipid in medium formulated with 5% of animal fat and 2.5% corn steep liquor yielded the highest reduction in surface tension (from 50 to 28 mN/m). Their result suggested that animal fat alone did not support high microbial growth and hence affecting the biosurfactant production as compared to the addition of corn steep liquor into production medium. The fatty acid composition of the animal fat substrate sourced from a bovine processing plant had been reported to contain higher composition of palmitic acid (26.40%) and oleic acid (24.16%) which could be the main reason affecting the biosurfactant production. On the contrary, *P. aeruginosa* was capable to consume 80% of the initial amount of oleic acid used and left a final residual concentration of about 8% to produce a maximum growth of 2.3 × 10^8^ of cells/mL after 3 days of incubation period [68].

As for fat containing a much higher fatty acid content, a comparative study on biosurfactant production by *P. aeruginosa* PAO1 was carried out on palm fatty acid distillate (PFAD), which is a by-product from a crude palm oil refinery plant having more than 70% of free fatty acids against glucose as the carbon components formulated into peptone-glucose ammonium salt (PPGas) medium [69]. The biosurfactant produced from 20–100 g/L of PFAD was in the range of 0.38–0.43 g/L, which was almost similar to the PPGas with glucose-containing medium (0.36 g/L). At higher concentrations of PFAD, biosurfactant production did not show any significant difference. This was mainly due to the low solubility of PFAD in the culture medium and as such, causing gross heterogeneity in the cultivation system. Therefore, regardless of the concentrations of PFAD being added, inadvertently there was a threshold amount of this substance able to dissolve in the medium which were indifferent in all runs. One of the advantages of using PFAD was that foam formation could be hampered during cultivation process due to the presence of free fatty acids which acts as an antifoaming agent [70]. PFAD can be a promising substrate for biosurfactant production and as to our present knowledge, it was the first published work on utilizing PFAD as substrate in biosurfactant production by *Pseudomonas* sp.

The third type of carbon source refers to hydrocarbon. Hydrocarbon is usually supplied in liquid form to biosurfactant-producing microorganism. For instance, isolated *P. aeruginosa* was produced biosurfactant which reduced the surface tension of culture to 30 mN/m from 65 mN/m within 3 days of utilizing hexadecane [71]. About 2.1 g/L of biomass was obtained after 11 days of incubation and nearly 70% of hexadecane was degraded after a 7 day incubation period which complemented a concurrent increase of biomass and biosurfactant produced. On the other hand, various strains of *P. aeruginosa* isolated from petroleum-contaminated soil in Assam, India were grown in various polycyclic aromatic hydrocarbon (PAH) substrates like phenantrene, fluorine, and pyrene [72]. The medium with mixtures of fluorine and phenantrene as carbon sources showed higher biosurfactant production by *P. aeruginosa* MTCC7815 (0.45 g/L) and MTCC7814 (0.38 g/L). The biosurfactant produced can actually increase the solubility of the hydrocarbon to be consumed by the microorganisms with the purpose of reinforcing their metabolism. Biosurfactants produced were identified as lipopeptide and protein-starch-lipid complex which are able to reduce surface tension from 72 mN/m to 35 mN/m of pure water. Last, but not least, is the utilization of diesel oil as the substrate, whereby *Aeromonas* sp. strain A2 could produce 0.067 g/L of biosurfactants after 7 days of incubation with 75% of the supplemented diesel oil had apparently been consumed [73]. Evidently, most of the biosurfactant can be produced by using all three types of carbon sources listed above. However performance-wise, cultivating microorganism which fed on oil and fat may be more favored over the rest. This might be due to the existence of a hydrophobic component in the substrate that will induce the production of hydrophobic moiety of biosurfactant though this is highly dependent on the behavior and metabolism of microorganism itself.

Pure substrates like glucose (Sigma, USD 64.75/kg), sucrose (Sigma, USD 91.68/kg), and glycerol (Sigma, USD 163.16/kg) are good candidates as carbon sources for various biosurfactant producers. However, wastes generated from industries and processing plants could also be potential candidates as carbon source for biosurfactant production (Table 2) as it is more economic and abundantly available compared to the pure substrates mentioned above, which may lead to food competition concerns. However, the variation of complex substrate’s composition between batches should be expected as its characteristic is fully dependent on the process and the raw material used. In particular, biosurfactant produced by *P. fluorescens* grown in media containing a mixture of natural manipueira (cassava flour wastewater) and nutrient broth reduced the surface tension of water to 59 mN/m from 80 mN/m compared to the media containing decanted manipueira [74]. Other agricultural waste like cashew apple juice (CAJ) was also utilized in biosurfactant production by *Acinetobacter calcoaceticus*. CAJ is rich in carbohydrates, fibers, vitamins, and minerals salt, that turn it into an interesting and inexpensive (USD 0.30/kg) substrate in the biosurfactant field [75]. Molasses that contains high amounts of sugar has been seen as one of the economical carbon sources (USD 0.10/kg) [76] for *P. aeruginosa* GS3 to grow and to produce biosurfactant. 

In term of performance, biosurfactants produced by *Deinococcus caeni* PO5 reduced the surface tension of the culture supernatant from 67.0 to 25.0 mN/m after 87 h of cultivation when 40 g/L of jackfruit seed powder and 1 g/L of commercial monosodium glutamate were used as the carbon and nitrogen sources, respectively [92]. Other than that, a newly discovered bacterium, *Lysinibacillus chungkukjangi* produced biosurfactant which reduced the surface tension of the media to 27.9 from 72 mN/m when the bacterium was grown on rice bran (by-product of rice milling) [93]. Rice bran contained high amounts of carbohydrates with 5% of bran which contained 12–18.5% oil [94]. In terms of price, the agricultural and industrial processing of waste like corn steep liquor (USD 0.46/kg), baggase (USD 0.04/kg), rice husk (USD 0.08/kg) are much cheaper compared to the pure substrate and more economical to be used as feedstocks. Besides that, utilization of novel substrates like vineyard pruning waste allowed *L. paracasei* to produce a biosurfactant with the highest surface tension reduction of water (27.3 mN/m) when grown in lactose-based medium [95].

### 4.2. Nitrogen Sources

Nitrogen is also required for microbial growth and production of certain primary and secondary metabolites [1]. The type of nitrogen existing in the production medium will affect the biosurfactant by microorganisms [96]. There are two types of nitrogen sources; organic and inorganic nitrogen. The two can be differentiated clearly based on the unit structure present in them. The unit structure for organic nitrogen will be in molecules such as yeast extract, meat extract, tryptone, or peptone, while inorganic nitrogen will have unit structures that consist of positive and negative ions like ammonium nitrate (NH_4_NO_3_). Organic nitrogen may also contain some carbon component and had been reported to significantly support cell growth and polysaccharides formation as compared to inorganic nitrogen [97] whereas nitrates, ammonia, and amino acids had been the nitrogen sources of choice for a few strains of *P. aeruginosa* [98]. 

Table 3 summarizes some of the organic nitrogen sources used in the production medium of biosurfactants by various microorganisms. Apparently, yeast extract has been widely chosen in many studies. It has been reported that a better emulsification index can be achieved when using a complex structure of the nitrogen source in the production medium, but it could become economically irrelevant in the MEOR (Microbial Enhanced Oil Recovery) process [99]. Nevertheless, Fooladi et al. [52] suggested that, while it uses can increase the concentration of biomass produced, somehow the substance showed less ability to reduce the surface tension as compared to other complex nitrogen sources. For instance, *L. paracasei* ssp. paracasei A20 favored yeast extract as the most important factor for bacterial growth and followed by meat extract, whereas peptone seems to be the least important factor when a medium containing a mixture of two different nitrogen sources was used to produce biosurfactant [100]. Most investigations on the different type of organic nitrogen sources would have fixed their concentrations used in the cultivation process. As such, an outright performance comparison of the cultivation outcome might be a little inaccurate since the true amount of nitrogen content, even for sources of similar types might differ for each batch or manufacturer, thus requiring the correct referral from the product datasheet.

It is far easier to determine the amount of nitrogen supplied in inorganic form based on their chemical formula and molecular weight. Table 4 represents some of the inorganic nitrogen sources in tandem with their exact nitrogen count in the culture medium from previously known bodies of work on biosurfactant production. It seems that most microorganisms in the list show preference towards nitrate-based nitrogen sources (NH_4_NO_3_ and NaNO_3_) for biosurfactant production compared to others. Perhaps, this attributes to the high nitrogen content readily available in these chemical solutions even when supplied in lower concentration. Microorganisms will first reduce nitrates to nitrite before turning it into ammonium. Then, ammonium is assimilated to form glutamate by glutamate dehydrogenase or to form glutamine by glutamine synthetase. l-glutamine 2-oxoglutarate aminotransferase will cause glutamine and α-ketoglutarate to be converted into glutamine. However, the formation of the lipid moiety rather than the sugar moiety in rhamnolipid is the rate-determining factor and various reports have shown that rhamnolipid can be produced more effectively in nitrogen-limiting conditions [70,110]. The production of biosurfactants often occurs when the nitrogen source is depleted in the culture medium during the stationary phase of cell growth. There is a possible inhibitory effect on the bacterial metabolism due to a likely nutrient transport deficiency as nitrate first undergoes nitrate reduction simulation to ammonium and then it is incorporated by glutamine-glutamate metabolism [111]. 

Others than the above nitrogen sources, there were few previous works utilizing waste materials to replace manufactured nitrogen sources for lowering the production cost. For instance, *P. aeruginosa* OG1 make used of chicken feather peptone (CFP) as the nitrogen source to yield maximum biosurfactant concentration (7.2 g/L). CFP contains high amounts of protein, ash, and nitrogen, low fat content, and various amino acids at different concentrations, especially alanine, leucine, glutamate, glycine, serine, and proline which make the most suitable nitrogen sources compared to yeast extracts, tryptone and peptone [117]. Other than being a substrate, corn steep liquor also had been used as nitrogen source together with NaNO_3_ for *P. aeruginosa* MR01 to produce 24 g/L of biosurfactant while employing soybean oil as the substrate [118]. Corn steep liquor is very rich in sugars (mainly starch, but also some glucose), soluble proteins (including peptides and amino acids), minerals (potassium, calcium, and magnesium) which make it a potential nitrogen source in biosurfactant production [119].

### 4.3. C/N Ratio

Carbon to nitrogen ratio (C/N) is the term to describe the relationship of carbon and nitrogen proportion needed in the production medium of biosurfactant by particular microorganisms. The C/N ratio required by microbial cultivation depends on the different types of microorganisms used, carbon and nitrogen type, culture conditions, and the desired product [120]. A previous study proved that nitrogen limitation conditions can cause the microorganisms to yield higher biosurfactant production and allows for alteration of the product’s composition to ensue [121]. High C/N ratios (i.e., low nitrogen levels) restrict bacterial growth, favoring cell metabolism towards the production of metabolites. For instance, when supplementing glycerol and NaNO_3_ as carbon and nitrogen sources on isolated *P. aeruginosa*, the optimal yield of biosurfactant (Y_P/S_) and bacterial cells (Y_P/X_) was found to be 0.13 g/g and 0.70 g/g, respectively, which corresponded to the C/N ratio of 55 [122]. Likewise, another isolated *P. aeruginosa* LBM10 produced an optimum biosurfactant concentration (1.42 g/L) when cultured in a nitrogen limiting condition (C/N ratio of 100) as compared to a C/N ratio of 22 (0.94 g/L) with soybean oil (carbon source) and NaNO_3_ (nitrogen source) being used in the production medium [123]. Both findings were using the same nitrogen source and same species of microorganism which led to maximum biosurfactant production under the nitrogen limiting conditions.

However, in most of the previous works, the common suitable C/N ratio for typical biosurfactant producer, *Pseudomonas* sp. is between 6 and 13 (non-nitrogen limiting condition) which seems contradictory to the previous statement [124,125,126,127]. For example, *P. aeruginosa* RS29 produced the highest biosurfactant (0.80 g/L) and maximum surface tension reduction (63.2 to 27.2 mN/m) when a C/N ratio of 12.5 was used. A higher C/N ratio (17.5 and 22.5) and lower C/N ratio (2.5 and 7.5) were tested and had caused a 25–39% reduction of biosurfactant production [128]. Other than that, higher biosurfactant production with maximum surface tension reduction can be seen when *P. fluorescens* Migula 1895-DSMZ was cultivated in a C/N ratio of 10 compared to 30 and 50 when olive oil and NH_4_NO_3_ were used as the carbon and nitrogen sources, respectively [129]. Hamzah et al. [130] suggested that there is a possible inhibitory effect occurred on *P. aeruginosa* UKMP14T when a C/N ratio of more than 20 was used. Therefore, the optimal biosurfactant performances which reduce surface tension from 47.43 to 30.6 mN/m at a C/N ratio of 14 were observed with glycerol and (NH_4_)_2_SO_4_ as the carbon and nitrogen sources, respectively. However, *P. nitroreducens* achieved the maximum biosurfactant production (5.5 g/L) when a C/N ratio of 22 was used and more than 18% of biosurfactant production was depleted when using other C/N ratios with glucose and NaNO_3_ acting as the carbon and nitrogen sources, respectively [111].

For other genus of microorganisms, *B. subtilis* strain showed that the increment of surface tension reduction could be observed at a lower C/N ratio condition (3 and 9) inversely proportional with the agitation speed used. At a C/N ratio of 15, it caused slight reduction in their performance in term of surface tension reduction even with the increased agitation rate where crystal sugar and NH_4_NO_3_ act as carbon and nitrogen sources, respectively [131]. This is further being supported when *B. subtilis* SPB1 produced the maximum amount of biosurfactant using 5 g/L of urea as organic nitrogen source and applying a C/N ratio of 7 with ammonium chloride as the inorganic nitrogen source [49]. In addition, *B. pumilus* 2IR yielded the maximum amount of biosurfactant (0.72 g/L) when potassium nitrate and glucose were used as a nitrogen and carbon sources, respectively, with a C/N ratio of 12. *Bacillus* sp. BMN 14 worked the best under a C/N ratio of 12.4, with a decrease in surface tension of up to 27 mN/m compared to other C/N ratios (10.6 and 17.51) with glucose and NH_4_NO_3_ utilized as the substrate and nitrogen source, respectively [132]. From the above study, most cases for *Bacillus* sp. preferred to have a lower C/N ratio to yield higher biosurfactant production.

In most cases, it seems like *Yarrowia lipolytica* showed an almost similar performance as *Bacillus* sp. as it achieved maximum surface tension reduction (19.0 mN/m) when a C/N ratio of 12 was used compared to lower or higher C/N ratios [133]. Others findings involved *Aureobasidium pullulans* YTP6-14 which acquired maximum surface tension reduction of up to 38.4 mN/m when using a C/N ratio of 300 compared to a C/N ratio of 100 and 200 with glucose and glycerol as carbon sources and NH_4_NO_3_ as the nitrogen source, respectively [134]. Higher amounts of carbon source are required as NH_4_NO_3_ provides two times the nitrogen source more than NaNO_3_ which can explain this outcome. *Virgibacillus salarius* attained maximum emulsifying activity (85%), and minimal surface tension (29 mN/m) at a C/N ratio of 30 (frying oil as carbon source; urea as nitrogen source) compared to other C/N ratios (10, 20, 40, 50, 60, and 70) [135].

### 4.4. Minerals

Minerals can be classified into two groups: macronutrient minerals and micronutrient minerals (trace elements). Potassium (K), calcium (Ca), magnesium (Mg), and iron (Fe) serve as macronutrient minerals in medium formulation which are important to balance the cell wall communication and aid in the protein synthesizing mechanism [136]. However, these metal ions can become an intracellular threat when present in excess. In the growth process, a metal ion acts as regulator for the production of physiologically active materials like a biosurfactant. Previous work proved iron, manganese, and magnesium to be cofactors of enzymes involved in the synthesis of surfactin by *B. subtilis* [27]. In this work, corn steep liquor (CSL) was utilized as the carbon source and the surfactin concentration was increased up to 4.8 g/L at optimum concentrations of these metal ions. They were supplemented simultaneously into the production medium. 

Potassium dihydrogen phosphate, KH_2_PO_4_ and dipotassium hydrogen phosphate, K_2_HPO_4_ are usually added into the production medium to maintain the desired pH throughout the cultivation process. For the buffering system, only phosphate ion serves as a pH regulator while potassium ion will be a source of energy as it is the major intracellular cation in bacteria [137]. Another study opted for potassium chloride (KCl) as the source of potassium in the medium containing glucose and alkane as the carbon sources for *Sphingobacterium detergens* [138]. It was suggested that potassium ion might be substantial for regulating ribosomal structures so that high amount of potassium ion should be present in growing bacteria [139]. 

Calcium works as a common mediator in signal delivering processes from the cell surface into the intracellular of microorganisms [140]. In biosurfactant production, calcium is usually supplied in the form of chloride or hydrated chloride salts, mostly in a concentration of 0.1 g/L for biosurfactant production from *P. aeruginosa* [112] and less than 0.02 g/L when producing glycolipid from *B. megatarium* [115] which goes to show that a tiny amount of Ca^2+^ ions are still required in the production medium. Both potassium and calcium ions play a crucial role in balancing the osmotic pressure and controlling the cell’s membrane potential which can prevent the lysis of the cell in the medium [141].

In order for ATP, which is the origin of the cell’s energy, to be active it would necessitate a bonding with a magnesium ion. This interaction is called Mg–ATP and the amount to be used will increase upon higher metabolic activity detected, and thus consequently increases in the rate of ADP and magnesium released [142]. Magnesium ion as typically supplied in the form of magnesium sulphate (MgSO_4_) and is mostly around 50 times higher than the concentration of calcium ion used in the production media of biosurfactants [124,128,143]. A study on the effect of individual metal ions towards growth and biosurfactant production points to significant influence of magnesium over other metals. When *Halobacterium salinarum* (HS) medium was supplemented with 5 mM of MgSO_4_, MnSO_4_, ZnSO_4_, CaCl_2_, FeSO_4_, and CoCl_2_, separately, *Halobacterium salinarum* J1 was able to obtain three-fold growth against controlled basal medium and doubled against the second best metal supplement (ZnSO_4_) and biosurfactant able to reach 68% of the E24 index [144].

Iron is a very popular cofactor in metabolism of various microorganisms. Most formulations will utilize iron in the form of Fe^2+^ or Fe^3+^ ions depending on the iron uptake mechanism of the microbe itself. Fe^2+^ is usually soluble in water and can be easily used by the microorganism compare to Fe^3+^. Most of the formulations in MSM medium will use Fe^2+^ supplied by FeSO_4_.7H_2_O [120,145] while Bushnell-Haas medium utilized FeCl_3_.6H_2_O to contribute Fe^3+^ in the production medium [77]. Works by Guerra-Santos et al. [146] recorded that the limitation of magnesium, calcium, and potassium might enhance rhamnolipid production by *P. aeruginosa*. 

On the other hand, addition of some micronutrient minerals or trace elements could have positive effect on biosurfactant production. Trace elements are chemical elements that are necessary for the microorganisms in amounts that are less than 0.1% of the total working volume. The specific requirements of trace elements are depending on the microorganism itself, but the most popular trace elements used by biosurfactant producer are zinc (Zn), copper (Cu), boron (B), molybdenum (Mo), and cobalt (Co). Makkar and Cameotra [57] added different concentrations of trace elements (0, 1, 2, 4, 6, 8, and 16 mL/L) into the production medium of the biosurfactant for *B. subtilis* MTCC 2423. The results demonstrated that the biosurfactant produced declined from a maximum value (1.30 g/L) when either more or less than 1 mL/L of the trace elements were used. However, the biomass had increased to more than 1.5 g/L when higher than 4 mL/L of trace elements were supplemented into the production medium. Based on this finding, the trace element limiting condition might cause overproduction of biosurfactants. This was further supported by Kiran et al. [147] demonstrating that further increments of FeSO_4_ and MgCl_2_ used from the original concentration (0.1 mM) drastically inhibit the biosurfactant production. More examples of trace elements used for biosurfactant production can be found in Table 5 along with their producer.

In some other cases, using the same trace elements with similar concentrations for different microorganisms might lead to wide differences in biosurfactant produced. For example, *B. megaterium* produced biosurfactant nearly 10 times higher than *P. aeruginosa* RS29 when cultivated in medium containing the same composition of trace elements [115,124]. It probably happened due to the different requirement levels by different microorganisms. Besides that, the addition of ethylenediaminetetraacetic acid (EDTA) into the production medium will induce the secretion of both moieties of the active compound in the biosurfactant. With the presence of this active compound, the function of the biosurfactant can be completed either by increasing the production of water soluble substrate or by solubilizing the nonpolar hydrocarbon substrates in production media [149]. EDTA which is a versatile chelating agent can potentially aid in forming complexes with metal ions like Fe^3+^, so that the ion can be gradually utilized by the microorganisms [25]. It can form four or six bonds with a metal ion, and chelates with both transition-metal ions and main-group ions to prevent them from precipitating in the medium.

### 4.5. Vitamins

Vitamin is the organic compound required in minute amount but was still necessary for the metabolic process for all organisms. Vitamins are rarely added on purpose since they are readily available in some of the natural medium components. Folic acid is one example of a vitamin that exists in the synthetic form of the water soluble B-vitamins which helps in synthesizing the nucleic acid for the microorganism to build up their DNA and is being added in small quantity into the medium. There is another type of vitamin B-complex, namely thiamine HCl, which is essential for the carbohydrate metabolism to digest the oily substrate in biosurfactant production. 

In terms of practicality, it is quite uncommon to add vitamin directly into the medium since the price of pure vitamin is expensive to begin with. Nonetheless, Qazi et al. [150] tried using vitamin B2 (1.0 g/L) as a nitrogen source to culture *P. putida* SOL-10. It was found that the growth performance was actually comparable to using sodium nitrite (NaNO_2_) as well as providing similar reduction in surface tension (35 mN/m). Other than that, beetroot waste was considered as a good potential substrate for biosurfactant production by *B. licheniformis* STK 01 [18]. Besides being rich in major nutrients (88% water, 1.2% protein, and 9.3% carbohydrates), it has all the mineral elements and essential trace amounts of carotene, thiamine, riboflavin, niacin, biotin, and vitamins C, E, B1, B2, and B12 [151]. *B. subtilis* MZ-7 was capable to grow on readily formulated pharmamedia commercial medium which contained significant amounts of vitamins such as carotene (<0.001 g/L), ascorbic acid (0.032 g/L), thiamine (0.004 g/L), riboflavin (0.005 g/L), niacin (0.083 g/L), choline (3.27 g/L), and pantothenic acid (0.012 g/L) with no carbon source provided and the microbe was capable of generating 0.22 g/L of surfactin [152]. Bayoumi et al. [153] managed to quantify the optimum vitamin concentration required by different microorganisms as presented in Table 6.

### 4.6. Metabolic Regulators

The microorganisms consume nutrients supplied into the production media for growth and metabolite production through various metabolic pathways. So, it is important for the production medium to contain essential elements that can aid in regulating those metabolic pathways. Based on previous works, some components other than those mentioned above added into the production medium can regulate the metabolism of microorganisms either by increasing or lowering biosurfactant production. Three important metabolic regulators, i.e., inhibitor and inducer which can influence the metabolism of microorganisms to produce biosurfactants were discussed below.

#### 4.6.1. Inhibitor

Some chemical compounds are prohibited from being added into the medium under any circumstances, or at least at certain concentrations since them can actually inhibit the production of biosurfactant or the growth of the cell. A medium containing hydrophilic substrates is commonly used compared to a medium with hydrophobic substrates as it aids in oxygen solubility into the production medium. But, some non-saccharide water-soluble substrates like ethanol, are well-known to inhibit the production of biosurfactants and to hinder the growth of microorganisms [154]. While this may be true, significant amounts of rhamnolipid (1.2 g/L) accompanied with a cell dry weight of 0.63 g/L were produced by *P. aeruginosa* when ethanol (30 g/L) was featured as the carbon source [105]. Other than that, antibiotics like carbenicillin (100 µg), chloramphenicol (30 µg), penicillin (10 UI), tetracycline (30 µg), ampicillin (10 µg), streptomycin (10 µg), and erythromycin (15 µg) were added into nutrient agar to grow *Rhodococcus ruber* and *R. erythropolis* to determine their level of antibiotic resistance. The result showed no sign of growth after these antibiotics were added into the production medium having diesel and glycerol as the carbon source [155]. According to this result, it indicates that those antibiotics might become the inhibitor. However, in some cases it is possible for the penicillin to cause the increment of biosurfactant by altering the cell wall permeability or releasing the cell wall-bound surfactant into the medium. For example, *Streptococcus sanguin* released 50% more of lipoteichoic acid in the penicillin-induced culture medium compared to the spontaneous culture medium [156]. In brief, only at a certain level of penicillin used in culture mediums will cause the inhibition of biosurfactant production. Another known inhibitor for biosurfactant production is EDTA, which also depends on the concentration used. At low concentrations of EDTA used (0.25 g/L), a maximum amount of glycolipid (more than 1.5 g/L) was produced by isolated *Tsukamurella* sp. nov. while consuming sunflower oil as the carbon source [157]. Addition of lower concentrations of EDTA led to a more homogenous culture during cultivation. In cultures where a slightly higher concentration of EDTA was used (0.5 g/L), the growth of *P. aeruginosa* S and Y were dramatically hindered and followed with weak performance in hexadecane biodegradation [158]. In other words, improper concentration used for certain chemicals might cause inhibition of biosurfactant along with the growth of microorganisms. 

#### 4.6.2. Inducer

An inducer is a chemical compound or substance that promotes the synthesis of the desired product when added into the production medium. An inducer will be added along with the main carbon source in the production medium. An inducer was added in smaller amounts than the carbon source used, to be just enough as the starting energy to boost up the growth of microorganisms in lag phase. Once the inducer depleted, the sole carbon source will be utilized for further biosurfactant production. The most effective inducer in biosurfactant production is olive oil. For instance, the biosurfactant production was enhanced through the biodegradation of lindane (carbon source) by Basidiomycetes yeast, *Rhodotorula* sp. VITJzN03 with an addition of 2% olive oil (E24 index, 78%) as compared to lindane alone being used as the carbon source (E24 index, 29%) [159]. In this work, biosurfactant production was enhanced as olive oil increased the hydrophobicity of the mineral medium containing lindane. For yeast-like fungus *Aureobasidium thailandense* LB01, the highest biosurfactant production (139 mg/L) was achieved when the culture medium containing glucose (6 g/L) as the carbon source and olive oil mill wastewater (1.5%, *w*/*w*) as the inducer along with yeast extract (2 g/L) as the nitrogen source were used after 48 h of cultivation when compared to the medium containing glucose as the sole carbon source [160]. Other than that, the hydrocarbons group are also known as popular inducers to improve biosurfactant production [161]. For example, *R. erythropolis* MTCC 2794 exhibited 53.84% of E24 index when 10 g/L of sucrose (carbon source) and 3% (*v*/*v*) of toluene (inducer) were added into the production medium [162].

### 4.7. Salinity Level

Salinity of the culture medium could become one of the critical parameters needed to be optimized to show the stability level of the biosurfactants produced by particular microorganisms. Frequently, sodium chloride (NaCl) is used to establish a certain level of salinity that adjusts osmolarity of media for microbial growth. Kiran et al. [147] showed that marine endosymbiotic fungus *Aspergillus ustus* (MSF3) isolated from the marine sponge *Fasciospongia cavernosa* exhibited the highest E24 index at 3% of NaCl compared to other NaCl concentrations used in the production medium. In the case of bacteria, Rismani et al. [163] showed that the cell growth of *B. licheniformis* was affected by different concentrations of NaCl with optimal cell growth and maximum surface tension reduction observed at 2% NaCl. For yeast, *Trichosporon asahii* demonstrated the highest E24 index when 8–10% of NaCl was added into the production medium [164].

In most findings, bacteria are capable to assimilate to a certain range of salt concentrations which is generally not more than 5%. For examples, *Aeromonas* spp. isolated from the tropical estuarine water required 5% of NaCl in MSM medium containing crude oil as the sole carbon source to achieve the highest E24 index compared to a medium devoid of salt or those with salt concentrations higher than 5.0% [165]. *Bacillus* sp. isolated from oil reservoirs was capable to reduce surface tension effectively at 5% of NaCl in comparison with 1–15% NaCl addition [166]. Moreover, *Bacillus* sp. (E24 = 70%) and *Pseudomonas* sp. (E24 = 79%) yielded maximum biosurfactant in the presence of 0.2% (*w*/*v*) until 0.8% (*w*/*v*) of NaCl [107]. In the case of yeast, it showed that the production of biosurfactant by *C. albicans* No. 13 increased as the NaCl concentration increased to 5% but the biosurfactant production decreased gradually when higher concentrations of NaCl were used [167]. In addition, Saikia et al. [168] also showed that at a very low concentration of NaCl or without NaCl supplementation to the mineral medium, *P. aeruginosa* RS29 showed good production of biosurfactants. 

In contrast, higher concentration of NaCl up to 30% could be compromised by *B. licheniformis* BAS50 to reduce surface tension to 35 mN/m [169]. Further increment of salt concentrations was not done in this work. It had been revealed that the intracellular concentration of rhamnolipid might be directly proportional to the external concentration of NaCl which explained this situation. Therefore, any increment of rhamnolipid intracellular concentration will cause the biosurfactant activity to be enhanced followed by the reduction of fat globulli dissimilation or addition of cytoplasmic membrane retention [170]. To conclude, cellular efficiency will be reduced and viscosity for both aqueous and oil phase will be increased followed by an increment in osmotic pressure at higher NaCl concentration which promotes higher emulsification activity.

For instance, the function of synthetic surfactant was greatly affected at NaCl concentrations higher than 2% which became their drawbacks. Biosurfactants can overcome this weakness by having the ability to resist a higher salinity level [32]. For example, rhamnolipid produced by bacteria isolated from the Arabian Sea coast of Karachi had the potential to be used in bioremediation for oil spill as the isolates represent the naturally occurring halophilic bacteria surviving in heavily contaminated regions [171]. On the other hand, halophilic archaeon *Haloarcula* sp. IRU1 surviving in production medium that contained 25% of NaCl (olive oil and yeast extract as carbon source and nitrogen source, respectively) demonstrated the maximum E24 index reading of 42.5%. This biosurfactant could be the promising candidate for oil recovery process [172].

### 4.8. Water

Water is the main component of the medium since most of the biosurfactant production in the laboratory or industrial scales will be run under submerged cultivation. For this reason, water has the biggest role in the production medium compared to other nutrient components. It can dissolve a wide range of important molecules from as simple as salt and sugar molecules, to large molecules such as amino acids and proteins [173]. In this part of the review, water is highlighted as the universal solvent used to dissolve the above mentioned production medium’s components only for the growth of biosurfactant producing microorganisms and subsequently to yield biosurfactants. In most of the previous works, the type of water or water quality used in biosurfactant production is rarely being discussed. In laboratory scale, the distilled water which is the common form of pure water is usually being used. Distilled water offers free dissolved or suspended material that is also safe for the growth of desired microorganisms which allow them to produce the required product. For example, distilled water was used to dissolve all components for isolate S2 [174], *P. putida*, *B. megatherium*, *B. licheniformis,* and *B. subtilis* [175] to grow and to yield biosurfactant in laboratory scale. It does not affect the properties of the product generated by microorganisms due to its neutral properties. However, the cost of energy required for heating during distillation process has made it rather uneconomic for larger industries. On the other hand, the use of tap water might be less favorable due to the presence of dissolved materials like iron, sulfate, and zinc [176] in unknown quantity which might affect the color of medium and nutrient content in it. Moreover, tap water usually contains high amounts of chlorine for disinfection purpose in the processing of a clean water supply and that will harm the microorganisms once it presents in the fermentation medium. However, it can be used in the preliminary step for isolation of biosurfactant-producing microorganisms. Singh (2012) used tap water to mix with the soil sample for purpose to isolate biosurfactant producing microorganisms, L4 (*Bacillus* spp.) and L15 (*Pseudomonas* spp.) which produced 0.074 g/L and 0.071 g/L of glycolipid, respectively [177].

## 5. Conclusions

This review provides scientific information on nutritional factors involved in the production medium of a biosurfactant. Despite the advantages of biosurfactants, its industrial use is still limited due to the high costs involved in the production process. Alternatively, the usage of inexpensive carbon and nitrogen sources from agricultural wastes and by-products of the process might give economic benefit to this process. In this review, we have presented various metabolic pathways involved in utilizing few types of carbon sources (hydrophilic and hydrophobic groups) by microorganisms and the significant influences of different nutritional factors on the biosurfactants production. The nutritional elements essential for the production of biosurfactants are categorized into the respective macronutrients, micronutrients, and trace elements. These elements are the key factors involved in the establishment of a suitable production medium for the biosurfactant. Different components, as stated above, exhibited distinct functions at certain concentrations or ratios that would considerably affect the cell growth as well as biosurfactant production. However, the exact concentrations or ratio used in the production medium might not be the same for all microorganisms as they are highly dependent on the existing metabolic pathways involved in metabolizing those components in the microorganisms. Thus, it necessitates the correct selection or optimization by experimenters during the process of medium design. From the discussion put forth, this review might contribute to the future study on optimization or possibly on the scaling up of biosurfactant production.

## Figures and Tables

**Table 1:** Common types of carbohydrates being used as carbon source for biosurfactant productions.

Carbon Source	Conc. (g/L)	Microorganisms	Biosurfactant (g/L)	Biomass (g/L)	Ref.
Glucose	8	*B. subtilis*	1.1	2.2	[21]
	150	*Candida bombicola* NRRL Y-17069	95.4	-	[48]
	40	*B. subtilis* SPB1 strain	0.72	-	[49]
	20	*P. aeruginosa* UMTKB-5	2.72	1.4	[50]
	20	*P. aeruginosa* MTCC 7815	3.88	5.67	[51]
	30	*B. pumilus* 2IR	0.72	2.75	[52]
	40	*B. subtilis*	3.6	-	[53]
	10	*B. subtilis* DSM 10^T^	0.16	0.59	[54]
	40	*P. aeruginosa* TMN	0.3	2.9	[55]
Sucrose	10	*B. subtilis* strains #573	2.16	1.03	[56]
	20	*B. subtilis*	0.8	2.5	[57]
	20	*P. putida* MTCC 2467	1.3	2.3	[58]
	20	*B. amyloliquefaciens* MB199	1.34	-	[59]
Starch	30	*Klebsiella* sp. RJ-03	10.1	-	[60]

**Table 2:** Example of agricultural or industrial wastes utilized in biosurfactant production.

Waste (Carbon Source)	Conc. (g/L)	Microorganisms	Biosurfactant (g/L)	Biomass (g/L)	Ref.
Molasses	70	*P. aeruginosa* GS3	0.24	0.8	[77]
Corn steep liquor	100	*B. subtilis* #573	4.47	-	[27]
Cassava processing effluent	-	*B. subtilis* LB5a	3.0	-	[78]
Potato peels	20	*B. pumilus* DSVP18	3.2	-	[79]
Soybean oil waste	80	*P. aeruginosa* MR01	25.5	5.15	[80]
Canola oil refinery wastes	20	*P. aeruginosa* EBN-8 mutant	8.5	4.5	[81]
Corn stover hydrolysate + yellow grease	10	*C. bombicola*	52.1	8.5	[82]
Baggase + soybean oil	100		84.6	7.7	[83]
Sugar beet molasses	50	*P. luteola* B17	0.53	-	[84]
		*P. putida* B12	0.52	-	
Banana peel	250	*Halobacteriaceae archaeon* AS65	5.3	4.8	[85]
Hydrolyzed distilled grape marc	-	*L. pentosus*	0.005	-	[86]
Orange peel	30	*P. aeruginosa* MTCC 2297	9.18	-	[87]
	40	*B. licheniformis*	1.796	-	[88]
Rice husk	125	*Mucor indicus*	0.078	-	[89]
Durian seed powder	45	*Ochrobactrum anthropi* 2/3	4.10	4.84	[90]
Palm oil decanter cake	250		4.52	-	[91]

**Table 3:** Example of organic nitrogen sources used in biosurfactant production by different microorganisms.

Nitrogen		Carbon		Microbe	Scale	Biosurfactant (g/L)	Biomass (g/L)	Ref.
Source	Conc. (g/L)	Source	Conc. (g/L)					
Yeast extract	2.0	Corn oil	20	*C. ingens* CB-216	500 mL	5.6	24.0	[101]
	5.0	Safflower oil	100	*Torulopsis bombicola* ATCC22214	7 L	18.0	12.4	[102]
	1.0	Soybean oil	80	*C. antarctica* ATCC20509	-	46.0	28.4	[103]
	2.0	Canola oil	100	*C. lipolytica* UCP0988	250 mL	8.0	-	[104]
	5.0	Glycerol	30	*P. aeruginosa*	250 mL	2.7	1.9	[105]
	4.0	Corn oil	10	*P. putida*	250 mL	3.5	-	[106]
	3.0	Glucose	1	*Bacillus* isolate	-	2.56	3.20	[107]
Urea	3.0	Brown sugar	10	*B. atrophaeus* 5-2a	600 mL	0.78	0.99	[108]
	1.5	Metalworking fluid oil	50.6	*P. aeruginosa* ATCC 9027	-	4.4	-	[109]
Peptone	1.0	Soybean oil	100	*Candida* sp. SY 16	5 L	37.0	10.0	[70]

**Table 4:** Examples of inorganic nitrogen sources used in biosurfactant production by different microorganisms.

Nitrogen			Carbon		Microbe	Scale	Biosurfactant (g/L)	Biomass (g/L)	Ref.
Source	Conc. (g/L)	Nitrogen Count (g/L)	Source	Conc. (g/L)					
Ammonium nitrate, NH_4_NO_3_	4.0	1.40	Palm oil	20	*P. aeruginosa* A41	-	6.58	-	[112]
	10.0	3.50	Soybean oil residue & glutamic acid	60 & 10	*C. lipolytica* UCP 0988	-	8.0	11.0	[113]
	1.0	0.35	Sodium acetate	20	*Bacillus* sp.	-	2.4	2.0	[114]
Sodium nitrate, NaNO_3_	2.0	0.33	Glucose	20	*P. nitroreducens*	250 mL	5.46	-	[111]
	6.0	0.99	Glycerol	30	*P. aeruginosa* UCP0992	500 mL	5.5	4.0	[6]
	14.0	2.30	Crude oil	20	*B. megaterium*	500 mL	3.58	1.4	[115]
	3.0	0.49	Glucose	1	*Pseudomonas* isolate	-	2.20	2.40	[107]
Ammonium sulfate, (NH_4_)_2_SO_4_	3.0	0.63	Sucrose	20	*B. subtilis*	1 L	0.20	0.8	[57]
	1.0	0.21	Glucose & fructose from cashew apple juice	10 & 8.7	*B. subtilis*	250 mL	0.123	-	[62]
	0.4	0.09	Pyrene	0.1	*Paenibacillus dendritiformis* CN5	250 mL	6.0	-	[116]
Potassium nitrate, KNO_3_	3.0	0.42	Glucose	30	*B. pumilus* 2 IR	1 L	0.72	3.46	[52]

**Table 5:** Some trace elements used in biosurfactant production by different microorganisms.

Microorganisms	Trace Elements (g/L)					Biosurfactant (g/L)	Ref.
	Zn	Cu	Mo	B	Mn		
*Bacillus* sp.	2.32	1.0	0.39	0.56	1.78	2.0	[114]
*P. nitroreducens*	0.005	0.071	0.015	0.015	0.2	6.0	[111]
*P. aeruginosa* PTCC1637	0.29	0.25	-	-	0.17	12.5	[148]
*P. aeruginosa* RS29	0.7	0.50	0.06	0.26	0.50	0.80	[124]
*B. megaterium*	0.7	0.50	0.06	0.26	0.50	7.8	[115]
*V. salarius*	0.29	0.25	-	-	0.17	2.8	[135]

**Table 6:** Example of different type of vitamins used to grow different microorganism [153].

Microorganisms	Substrate	Type of Vitamins	Concentration (g/L)	Surface Tension (mN/m)
*P. illinoisensis*-21	Crude oil	Folic acid	0.2	39
*B. subtilis*-27		Thiamine HCl	0.2	40
*Bordetella hinizi*-DAFI		Folic acid	0.2	42
